# Structural Determinants of Oligomerization of the Aquaporin-4 Channel*

**DOI:** 10.1074/jbc.M115.694729

**Published:** 2016-01-19

**Authors:** Philip Kitchen, Matthew T. Conner, Roslyn M. Bill, Alex C. Conner

**Affiliations:** From the ‡ Molecular Assembly and Organisation in Cells Doctoral Training Centre, University of Warwick, Coventry CV4 7AL,; the ‖ School of Biology, Chemistry and Forensic Science, Faculty of Science and Engineering, University of Wolverhampton, Wolverhampton WV1 1LY,; the § School of Life & Health Sciences and Aston Research Centre for Healthy Ageing, Aston University, Aston Triangle, Birmingham, B4 7ET, and; the ¶ Institute of Clinical Sciences, University of Birmingham, Edgbaston, Birmingham B15 2TT, United Kingdom

**Keywords:** aquaporin, cellular regulation, oligomerization, protein translocation, water channel

## Abstract

The aquaporin (AQP) family of integral membrane protein channels mediate cellular water and solute flow. Although qualitative and quantitative differences in channel permeability, selectivity, subcellular localization, and trafficking responses have been observed for different members of the AQP family, the signature homotetrameric quaternary structure is conserved. Using a variety of biophysical techniques, we show that mutations to an intracellular loop (loop D) of human AQP4 reduce oligomerization. Non-tetrameric AQP4 mutants are unable to relocalize to the plasma membrane in response to changes in extracellular tonicity, despite equivalent constitutive surface expression levels and water permeability to wild-type AQP4. A network of AQP4 loop D hydrogen bonding interactions, identified using molecular dynamics simulations and based on a comparative mutagenic analysis of AQPs 1, 3, and 4, suggest that loop D interactions may provide a general structural framework for tetrameric assembly within the AQP family.

## Introduction

The aquaporin (AQP)^3^ family of integral membrane proteins facilitate both osmosis and diffusion of small polar molecules through biological membranes. A wealth of medium-to-high resolution structural data for various family members (there are 48 AQP structures in the Protein Data Bank) all suggest that the AQP homotetrameric quaternary structure is highly conserved despite diversity in solute permeability, subcellular localization, and trafficking responses of individual AQPs. Early biochemical work, using carboxyl-amine fusion dimers (consisting of 1 wild-type unit and 1 unit lacking the cysteine residue required for mercurial inhibition), showed that monomers are the functional AQP units (1); numerous molecular dynamics simulation studies support this (2). Recent work has suggested that isolated AQP monomers are equally capable of facilitating water transport as those incorporated into a tetramer (3). Therefore it is not clear why AQPs retain this tetrameric structure. Regulation of AQP function by the formation of heterotetramers has been suggested for some plant AQPs (4). The fifth, central pore formed at the 4-fold axis of the tetramer has also been suggested to transport carbon dioxide (5) and cations (6), at least in mammalian AQP1. Trigger-induced relocalization of AQP-containing vesicles to the plasma membrane is a well established regulatory mechanism for AQPs; the best studied example of this is relocalization of AQP2 to the apical membrane of the collecting duct in the mammalian kidney in response to arginine vasopressin (also called anti-diuretic hormone). There is also evidence for similar mechanisms for other AQPs including AQP1 (7), AQP5 (8), and AQP7 (9). A naturally occurring AQP2 mutant (R187C) has also been reported that is unable to relocalize in response to arginine vasopressin or to form tetramers (10). Given the ubiquity of these regulatory responses across the AQP family and the conservation of the tetrameric quaternary structure, it may be that these trigger-induced relocalization responses involve interaction with proteins that only recognize the tetrameric form of AQPs.

Here we demonstrate that intracellular loop D of AQP4 forms vital homomeric interactions between AQP subunits that stabilize the tetrameric quaternary structure. We also show that loss of tetramerization does not affect single channel water permeability. Our data suggest that tetramerization is not required for AQP4 to be trafficked through the endoplasmic reticulum and Golgi to the plasma membrane, but that unlike wild-type AQP4, the non-tetrameric mutants are unable to relocalize to the plasma membrane in response to changes in local osmolality. Finally, based on loss and gain of oligomerization mutants of AQP1 and AQP3, we suggest that loop D-mediated inter-monomer interactions may control formation of the signature quaternary structure of the family.

## Experimental Procedures

### 

#### 

##### Expression Constructs and Mutagenesis

Human AQP4 cDNA cloned into pDEST47 (Life Technologies) was used as previously described (11). An untagged AQP4 construct was created from this by mutagenesis of the first two codons of the GFP linker peptide to stop codons. These were used as templates for mutagenesis following the QuikChange protocol (Stratagene). Mutagenic primers were synthesized by Sigma. Mutant plasmids were amplified in TOP10 *Escherichia coli* with 100 ng/ml of ampicillin selection. Plasmid DNA was purified using a Wizard Maxiprep kit (Promega) and diluted to 1 mg/ml for transfection.

##### Cell Culture and Transfection

HEK293 cells were cultured routinely in DMEM with l-glutamine (Sigma) supplemented with 10% (v/v) fetal bovine serum (Sigma) and without antibiotics in humidified 5% (v/v) CO_2_ in air at 37 °C. Cells were seeded into either tissue culture treated 6-well plates (Falcon) for Blue Native (BN)-PAGE and biotinylation or 35-mm FluoroDishes^TM^ (World Precision Instruments) for confocal microscopy. Cells were transfected (at 50% confluence) using polyethyleneimine (branched, average *M*_r_ ∼25,000, Sigma) as previously described (11). MDCK cells were cultured in the same conditions as HEK293 cells. Stable transfections were done using the neomycin resistance gene on the pDEST47 vector. Cells were transiently transfected as described above, then trypsinized and serially diluted into tissue culture plates after 24 h. Cells were treated with 700 μg/ml of G418 antibiotic for 2 weeks, with medium replaced every third day. GFP-expressing resistant colonies were picked using cloning cylinders (Sigma) and serially diluted. The lowest dilution that grew to confluence was used to generate a stable cell line. Cellular protein was subjected to SDS-PAGE and Western blotting as previously described (11), and the highest expressing clone was chosen for experiments. No endogenous AQP4 was detected in Western blots. Reduced G418 pressure (300 μg/ml) was used to maintain the stable cell lines after colony isolation. All cells were routinely tested for mycoplasma using the EZ-PCR test kit (Biological Industries), and all data reported are from cells that tested negative. HEK293 and MDCK cells both expressed only the M1 isoform of AQP4 from the wild-type AQP4 construct. This was confirmed by Western blotting comparing the wild-type construct to AQP4 constructs in which either the M1 or M23 translation initiation sites were removed, as previously described (11).

##### Confocal Microscopy

AQP4-GFP constructs were imaged in live HEK293 cells using a Zeiss LSM 780 confocal microscope with a ×63 1.4 NA oil immersion objective. GFP was excited using the 488-nm line of an argon laser, Venus using the 514-nm line and mTurquoise2 using a 405-nm diode laser. Hypotonic exposure was performed by adding 3 ml of ddH_2_O to cells in 1 ml of growth medium (280 to 70 mosmol/kg of H_2_O). Line profiles across the cell membrane and cytoplasm were extracted using ImageJ as previously described (7). Relative membrane expression was calculated from these profiles (3 profiles per cell and at least 3 cells per image) using in-house Matlab code. Fluorescence recovery after photobleaching (FRAP) was done using a circular bleaching 1-μm area of radius. Recovery curves were fitted to a single phase exponential recovery function and diffusion coefficients were calculated using the approach and equations of Kang *et al.* (12). Recovery curves were collected from 5 different cells on the same plate per experiment. FRET experiments were done using the sensitized emission methodology with the FRET signal corrected for donor emission in the acceptor channel and direct excitation of the acceptor, following van Rheenen *et al.* (13) and normalized to the acceptor emission to give an apparent FRET efficiency. The contrast of some images in the figures was adjusted manually using ImageJ to aid the eye. All analysis was performed on raw, unadjusted images.

##### Cell Surface Biotinylation

Cell surface amines were biotinylated using an amine-reactive biotinylation reagent that is not cell permeable (Thermo number 21328, EZ-Link Sulfo-NHS-SS-Biotin), and surface AQPs were detected using a neutravidin-based ELISA as previously described (11).

##### BN-PAGE

Transfected cells were lysed in ice-cold BN lysis buffer (1% (v/v) Triton X-100, 10% (v/v) glycerol, 20 mm bis-tris, 500 mm aminohexanoic acid, 20 mm NaCl, 2 mm EDTA, pH 7.0, 250 μl/well). The lysate was centrifuged at 21,000 × *g* at 4 °C for 10 min to remove insoluble material. The supernatant was collected and diluted 10-fold in Triton lysis buffer. 8% bis-tris-buffered polyacrylamide gels (0.75 mm) at pH 7.0 containing 66 mm aminohexanoic acid were used. Wells were topped up with cathode buffer (50 mm Tricine, 15 mm bis-tris, 0.02% (w/v) Coomassie G-250, pH 7.0). 10 μg of BSA was used as a molecular mass marker, giving bands at 66 and 132 kDa. Gels were run on ice at 100 V until samples entered the gel, then at 180 V until the Coomassie dye front reached the end of the gel.

##### Immunoblotting

BN-PAGE gels were destained with 40% (v/v) methanol, 10% (v/v) glacial acetic acid for 30 min, refreshing the destaining solution every 10 min. Gels were soaked for 30 min in 1% SDS in Tris-buffered saline, pH 7.4, at room temperature. Proteins were blotted onto PVDF membrane by wet transfer at 100 V for 1 h. Coomassie-stained BSA marker bands were marked onto the membrane using a felt-tipped pen. Membranes were blocked in 20% (w/v) Marvel-skimmed milk powder in 0.1% PBS-Tween for 1 h. Membranes were incubated overnight at 4 °C on a roller in rabbit anti-AQP4 antibody (Abcam, ab128906) diluted 1:5,000 or rabbit anti-GFP (Abcam, ab6556) diluted 1:10,000, both in 5 ml of 0.1% PBS-Tween. Membranes were washed in 0.1% PBS-Tween and incubated with donkey anti-rabbit HRP (Santa Cruz, sc-2313) diluted 1:10,000 in 20 ml of 0.1% PBS-Tween at room temperature for 1 h. HRP was detected on x-ray film using ECL reagent (Amersham Biosciences).

##### Statistics

For multiple comparisons, one-way ANOVA was used, followed by post hoc *t*-tests with the *p* values subjected to Bonferroni correction for multiple comparisons. All data are presented as mean ± S.E.

##### Simulations

Simulations were done using the GROMOS 53A6 forcefield (14) extended to include lipid parameters (15) in Gromacs version 4.5.5 (16). An AQP4 tetramer was generated according to the biological assembly entry in the AQP4 Protein Data Bank file 3GD8 (17). N and C termini of the protein were truncated in the structure so proteins were simulated with neutral termini. The AQP4 tetramer was embedded into 5 pre-equilibrated 1-palmitoyl-2-oleoyl-*sn*-glycero-3-phosphocholine bilayers using inflateGRO (18) and hydrated using Gromacs. Na^+^ and Cl^−^ were added to a final concentration of 100 mm. Equilibration was achieved by steepest gradient energy minimization, 100-ps NPT simulation with 1,000 kJmol^−1^ nm^−1^ restraints on protein heavy atoms followed by three 1-ns NVT simulations with 1000, 100, and 10 kJmol^−1^ nm^−1^ restraints on protein heavy atoms followed by 30-ns unrestrained simulation. A Nosé-Hoover thermostat (0.5 ps, 310 K) was used to maintain constant temperature and 2 Parinello-Rahmann barostats (2 ps, 1 atm) were used to maintain constant pressure with zero surface tension. 1.4-nm cut-offs were applied for dispersion and short-range electrostatic interactions. Long-range electrostatics were treated using particle mesh Ewald. Hydrogen bonds were identified using the H-bonds plugin of visual molecular dynamics (19) using 3 Å and 20° cut-offs. Hydrogen bond occupancy was calculated according to these cut-offs at 100-ps intervals along the 100-ns trajectories and averaged over the 4 monomers and 5 trajectories.

##### Calcein Fluorescence Quenching

Plate reader-based calcein fluorescence quenching was done following Fenton *et al.* (20). MDCK cells were plated into black-walled, clear-bottomed tissue culture treated 96-well plates (Greiner) at 50% confluence 24 h before the experiment. Cells were loaded with 5 μm calcein-AM in growth medium supplemented with 1 mm probenecid (to inhibit dye leakage) for 90 min. Cells were washed twice with HEPES-buffered growth medium supplemented with 1 mm probenecid, then covered with 75 μl of probenecid-supplemented HEPES-buffered medium. Fluorescence was read on a BioTek synergy HT plate reader with injector system. Each well was read continuously (*dt* = 50 ms) for 5 s, followed by injection of 75 μl of HEPES-buffered medium containing 400 mm mannitol to give a final concentration of 200 mm and an osmotic gradient of 200 mosmol. Fluorescence was read for a further 50 s. Normalized fluorescence values were converted to normalized volumes using a Coulter counter generated standard curve. Single-phase exponential decay functions were fitted and rate constants were taken as proportional to the membrane water permeability.

## Results

### Mutation of Loop D of AQP4 Reduces Tetrameric Assembly

**FIGURE 1.**
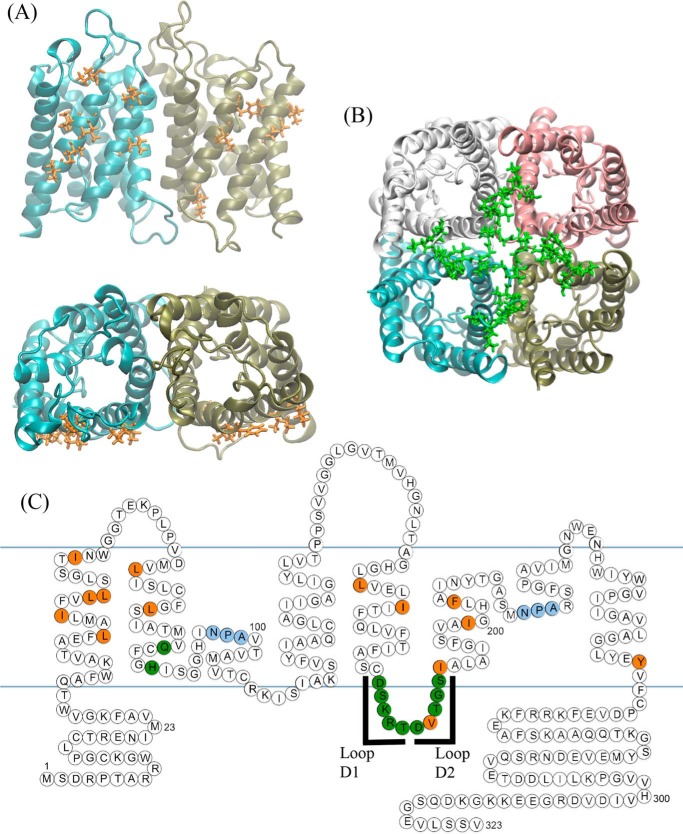
**Residues at the AQP4 tetrameric interface.** *A,* we identified hydrophobic residues (*orange*) in TMs 1, 2, 4, and 5 that formed inter-monomer contacts in the crystal structure and *B,* polar residues (*green*) at the bottom of TM2 and in the intracellular loop D. *C,* ball diagram showing the position of the identified residues in the primary sequences and secondary structural motifs of AQP4. Two regions of loop D were selected for compound mutation, which we denote loop D1 (^179^DSKRT^183^) and loop D2 (^184^DVTGS^188^). *Blue lines* represent the approximate position of membrane lipid headgroups. All residues are listed in Table 1.

**FIGURE 2.**
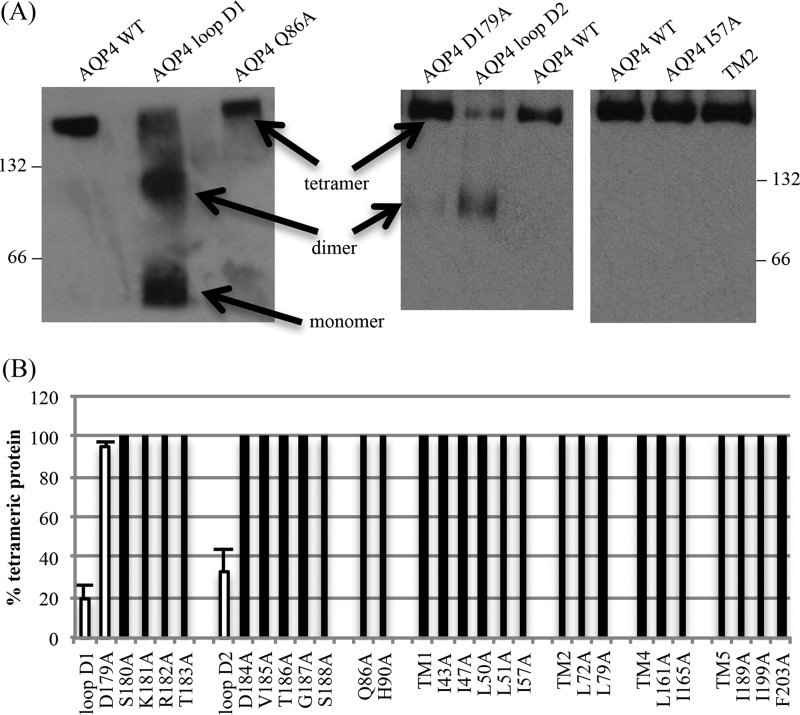
**BN-PAGE and Western blotting of AQP4 mutants.** *A,* representative Western blots following BN-PAGE of Triton X-100-solubilized AQP4 mutants, showing the effect of the loop D1 and loop D2 compound mutations, and a lack of effect of mutations on the transmembrane hydrophobic patch. *66* and *132* denote the positions of BSA molecular weight marker bands. The AQP4-GFP construct, including linker peptide, has a predicted molecular mass of 63.1 kDa. *B,* percentage of protein assembled into tetramers calculated using densitometry following BN-PAGE and Western blotting. Effective mutations are highlighted with *white bars*. Data are presented as mean ± S.E. from 3 experimental repeats.

Using the crystal structure of AQP4 (17), residues likely to form the tetrameric interface were identified, based on the physical distance between residues on adjacent monomers. Alanine substitution mutagenesis was used to investigate the contribution of these residues to oligomeric assembly. The identified residues were clustered into two regions. The first cluster comprises a patch of hydrophobic residues at the interfaces of TM1 and TM2 of one monomer with TM4 and TM5 of the adjacent monomer (Fig. 1*A*); the second cluster comprises 12 predominantly polar and charged residues forming intracellular loop D and the bottom of TM2 (Fig. 1*B*). 24 single point alanine-substitution mutants were made and six compound mutants were used to investigate the possibility of synergistic effects of several residues. There are two protein kinase A/C consensus sites in loop D (Ser^180^ and Ser^188^) so phosphomimetic mutations were also made (S180D and S188D). All mutants are listed in the left-hand column of Table 1. BN-PAGE followed by immunoblotting was used to assess the oligomeric state of all mutants expressed in HEK293 cells. Representative BN blots are shown in Fig. 2*A* and the effect of all mutations on oligomeric assembly and surface expression summarized in Table 1 and Fig. 2*B*. None of the hydrophobic residues in the hydrophobic patches had any effect on tetramer formation, either in isolation or in compound mutants. TM compound mutants consisted of simultaneous mutations of all residues identified within that transmembrane segment (*e.g.* TM1 denotes the simultaneous mutations I43A/I47A/L50A/L51A/I57A). Of the loop D single alanine mutants, only D179A had an effect on oligomeric assembly in isolation, and this effect was only minimal, with 95 ± 2% of the protein still assembled into tetramers. Unlike the hydrophobic cluster, the compound mutants of loop D had a clear effect on the ability of AQP4 to tetramerize. The two compound mutants, loop D1 (D179A/S180A/K181A/R182A/T183A) and loop D2 (D184A/V185A/T186A/G187A/S188A), caused reductions in oligomeric assembly: only 19 ± 4% of the loop D1 protein assembled into tetramers with both dimers (27 ± 5%) and monomers (53 ± 4%) being present, whereas the loop D2 protein predominantly formed dimers (67 ± 7%) with 33 ± 7% assembled into tetramers (*n* = 3).

**TABLE 1:** **Oligomerization state and surface expression of AQP4 mutants** Oligomerization state of all AQP4 mutants analyzed. Compound mutations are represented by a solid slash between the relevant point mutations, *e.g*. L72A/L79A. T = tetramer, D = dimer, M = monomer. All data are reported as mean ± S.E., *n* = 3.

Mutant	Oligomerization state	Surface expression
		% *of WT*
I43A	T	113.9 ± 5.1
I47A	T	95.8 ± 3.2
L50A	T	102.5 ± 4.1
L51A	T	96.1 ± 13.0
I57A	T	104.1 ± 4.1
TM1 (I43A/I47A/L50A/L51A/I57A)	T	86.9 ± 3.1
L72A	T	121.1 ± 25.6
L79A	T	88.9 ± 13.3
TM2 (L72A/L79A)	T	97.7 ± 5.6
L161A	T	91.9 ± 18.4
I165A	T	87.9 ± 3.6
TM4 (L161A/I165A)	T	85.4 ± 11.2
I189A	T	97.7 ± 5.9
I199A	T	96.2 ± 8.5
F203A	T	112.4 ± 13.3
TM5 (I189A/I199A/F203A)	T	93.3 ± 9.0
Q86A	T	118 ± 4.4
H90A	T	113.5 ± 4.1
D179A	T (95 ± 2%)	87.6 ± 19.4
	D (5 ± 2%)	
S180A	T	109.8 ± 4.5
S180D	T	99.7 ± 4.9
K181A	T	103.2 ± 4.5
R182A	T	96.1 ± 22.1
T183	T	105.6 ± 3.6
Loop D1 (D179A/S180A/K181A/R182A/T183A)	T (19 ± 4%)	88.5 ± 6.1
	D (27 ± 5%)	
	M (53 ± 4%)	
D184A	T	97.7 ± 8.8
V185A	T	102.2 ± 5.4
T186A	T	95.1 ± 7.5
G187A	T	106.7 ± 5.1
S188A	T	98.6 ± 3.2
S188D	T	104.1 ± 4.2
Loop D2 (D184A/V185A/T186A/G187A/S188A)	T (33 ± 7%)	83.1 ± 11.0
	D (67 ± 7%)	

### Surface Expression of Non-tetrameric Mutants

**FIGURE 3.**
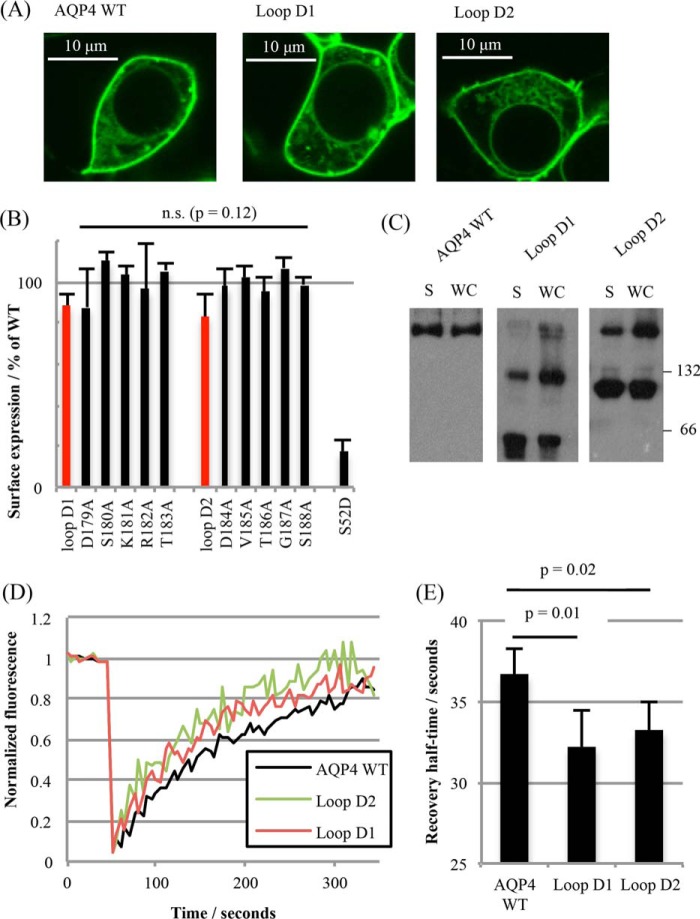
**Plasma membrane localization of non-tetrameric mutants.** *A,* representative fluorescence micrographs of HEK293 cells transfected with C-terminal GFP fusions of AQP4 WT and loop D mutants. *B,* surface expression of AQP4 mutants in HEK293 cells measured by cell surface biotinylation followed by a neutravidin-based ELISA. Loop D compound mutants are highlighted in *red*. The S52D mutant was used as a negative control for surface expression. *n.s*., not significant. *C,* reprsentative blots of AQP4 mutants subjected to BN-PAGE. *WC*, whole cell lysate; *S,* surface protein only, isolated by cell surface biotinylation. *D,* representative FRAP curves from photobleaching AQP4-GFP fusion proteins in HEK293 cells. *E,* average half-times of fluorescence recovery averaged over fits to 5 curves per experiment and 6 experimental repeats. All data are presented as mean ± S.E.

There was no significant difference in the surface expression of the loop D mutants compared with wild-type AQP4. Surface expression was assessed qualitatively by live cell confocal microscopy using GFP-tagged AQP4 mutant constructs and quantitatively by cell surface biotinylation. Fig. 3*A* shows representative confocal micrographs of HEK293 cells transfected with GFP fusion proteins of AQP4 wild-type, and the loop D1 and loop D2 mutants. Surface expression in transiently transfected HEK293 cells measured by cell surface biotinylation was not significantly different (*p* = 0.53, one-way ANOVA, *n* = 3) for either of the loop D compound mutants (D1 and D2) or the single alanine mutants (Fig. 3*B*). This suggests that the trafficking machinery is able to interact with non-tetrameric aquaporins. The cell surface biotinylation data are summarized for all mutants in the third column of Table 1.

### Mutants in the Plasma Membrane Exhibit Reduced Tetramerization

It was important to confirm the reduction in tetramerization of AQP4 molecules that had been constitutively trafficked to the cell surface and to rule out intracellular retention of dimeric/monomeric species or changes in detergent sensitivity caused by loop D substitutions. Several complementary biophysical techniques were used to address this.

#### 

##### Fluorescence Recovery after Photobleaching (FRAP)

Recovery curves were collected from 5 different cells per experimental repeat (*n* = 6). Representative fluorescence recovery curves are shown in Fig. 3*D*, along with the average recovery half-times. From these, diffusion coefficients were calculated: 5.2 ± 0.3 × 10^−3^ μm^2^ s^−1^ (AQP4 WT), 5.9 ± 0.3 × 10^−3^ μm^2^ s^−1^ (loop D1), and 5.8 ± 0.2 × 10^−3^ μm^2^ s^−1^ (loop D2). Both loop D mutant diffusion coefficients were significantly different from the wild-type (D1 *p* = 0.01 and D2 *p* = 0.02 by Student's *t* test following ANOVA, with *p* values subjected to Bonferroni correction). The FRAP data suggest reduced tetramerization for the surface-localized loop D mutants, although the increased diffusion coefficient could also be explained by the inability of these mutants to form a complex with a third party protein.

##### BN-PAGE of Biotinylated Cell Surface Protein

To complement these FRAP experiments, biotinylated cell surface proteins were isolated using neutravidin-coated plates, eluted by reducing the S-S bond incorporated into the biotinylation reagent (using 1% β-mercaptoethanol in BN lysis buffer) and subjected to BN-PAGE (representative blots are shown in Fig. 3*C*). Surface-localized mutant AQP4 molecules subjected to BN-PAGE had the same changes in tetramerization seen in whole cell lysates (*n* = 3).

##### Forster Resonant Energy Transfer (FRET)

**FIGURE 4.**
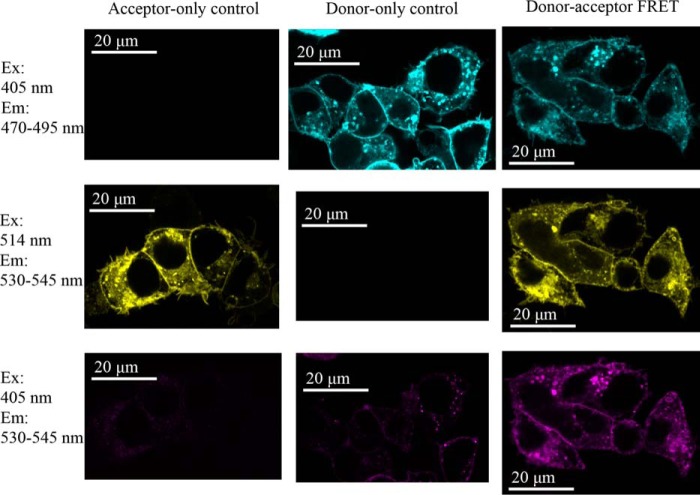
**A FRET biosensor for AQP4 oligomerization.** *A,* fluorescence confocal microscopy of live HEK293 cells transiently transfected with AQP4-Venus alone, AQP4-mTurquoise2 alone, and the two co-transfected. Excitation (*Ex*) at 405 nm is for Turquoise and 514 is for Venus. The contrast of these images has been manually optimized to aid the eye. All analysis was performed on raw, unadjusted images. *Em*, emission.

**FIGURE 5.**
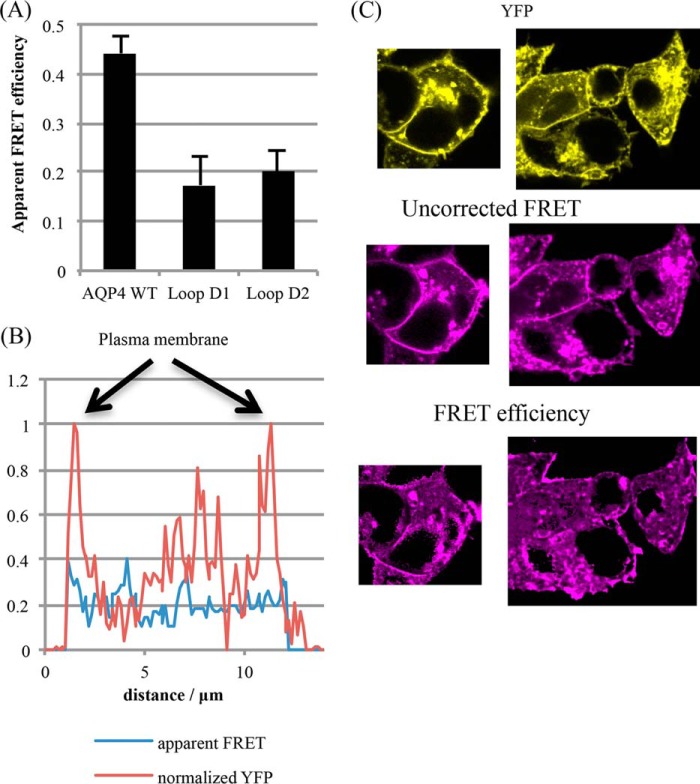
**Reduced FRET from AQP4 mutants.** *A,* average apparent FRET efficiencies for AQP4 wild-type and loop D1 and D2 mutants, calculated by normalizing the corrected FRET intensity to YFP intensity for each pixel. Five different areas on each plate were imaged per experimental repeat, *n* = 4. *B,* representative line scan across a cell, passing through membrane and cytoplasm and avoiding the nucleus. Whereas the YFP signal (*red*) shows clear peaks at the plasma membrane, the FRET efficiency (*blue*) does not. *C,* representative processed FRET efficiency images compared with unprocessed FRET and YFP. Whereas both YFP and the raw FRET show clear membrane signals, the FRET efficiency does not.

To complement the above analyses, AQP4 constructs tagged with Venus (a yellow fluorescent protein (YFP) derivative) and mTurquoise2 (a cyan fluorescent protein (CFP) derivative) were generated and co-transfected into HEK293 cells to form a FRET biosensor for homo-oligomerization in living cells. The wild-type AQP4 constructs gave a robust FRET signal with an average apparent efficiency of 44.2 ± 3.6% (Fig. 4). We were unable to measure any FRET in cells co-transfected with AQP1-Venus and AQP4-Turquoise despite high co-localization (data not shown), suggesting that the FRET interactions occur primarily within the AQP4 tetramers and not between tetramers that are transiently close together in the plane of the membrane. The probability of a particular donor molecule taking part in FRET is dependent on the number of acceptors within the Forster radius and vice versa. For CFP-YFP, the Forster radius is ∼5 nm (21). Based on the AQP4 crystal structure, the monomer-monomer center of mass separations are 2.8 (adjacent monomers) and 3.9 nm (diagonal monomers), respectively, so both would be expected to contribute to the FRET signal (assuming that the average separation of the C-terminal tails is similar). The average number of FRET pairings in a sample of co-transfected cells is therefore dependent on the level of AQP4 oligomerization. Both D1 and D2 compound mutants had a slightly larger than 2-fold reduction in FRET efficiency (to 17 ± 6 and 20 ± 4%, respectively, *p* = 0.003 and *p* = 0.005, *n* = 4) compared with the wild-type (Fig. 5*A*), suggesting that these constructs have a reduced propensity to oligomerize in live cells, further confirming that the changes seen in the BN-PAGE were not mediated by changes in detergent sensitivity. Furthermore, for the mutants, there was no difference in FRET efficiency between plasma membrane and intracellular membranes (Figs. 5, *B* and *C*), suggesting that the oligomerization state of these mutants is the same in all membrane compartments (*e.g.* Golgi, vesicles, plasma membrane). Taken together, these data suggest reduced tetramerization for the AQP4 loop D mutants in the plasma membrane of living cells.

##### Loop D Mutants Have Wild-type Water Permeability

**FIGURE 6.**
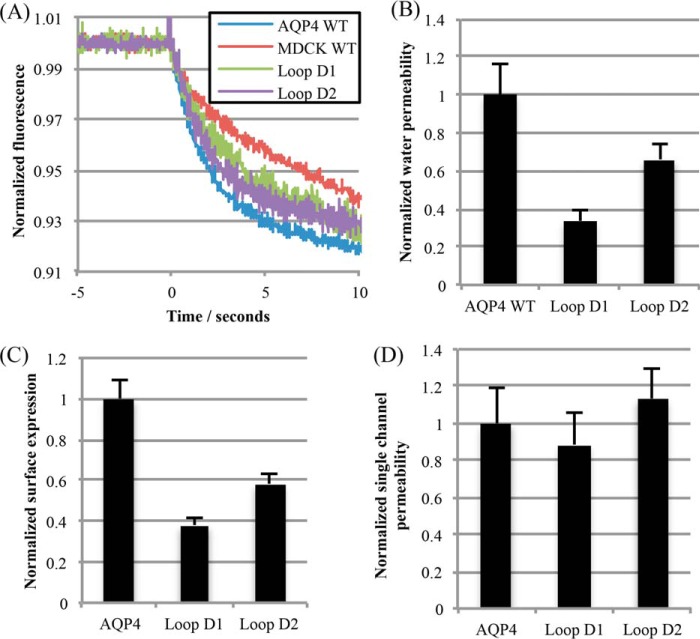
**Water permeability of non-tetrameric mutants.** *A,* representative calcein fluorescence quenching curves from stably transfected MDCK cells subjected to a 200 mosmol of mannitol osmotic gradient. *B,* water permeability of MDCK cells normalized to AQP4 WT-transfected MDCK cells. *C,* normalized surface expression of AQP4 constructs in the stably expressing MDCK clones used for water permeability measurements, measured by cell surface biotinylation. *D,* MDCK membrane water permeability normalized to surface expression, to give normalized single channel permeability. All data are presented as mean ± S.E., *n* = 4.

Both loop D compound mutants (D1 and D2) and wild-type AQP4 were stably transfected into MDCK cells to measure water channel function. Membrane water permeability of MDCK cells, measured by calcein fluorescence quenching (Fig. 6*A*), was increased 7-fold by stable expression of AQP4. Water permeability due to the transfected AQP was calculated by subtracting the permeability of untransfected cells (Fig. 6*B*); the resulting permeability was normalized to the surface expression measured by cell surface biotinylation to allow for differences in cellular expression due to differences in the position of chromosomal integration of the stably transfected gene (Fig. 6, *C* and *D*). After normalization to surface expression, no significant difference in permeability was observed between the loop D mutants and wild-type AQP4 (one-way ANOVA, *p* = 0.17, *n* = 4), suggesting that tetramerization of AQP4 is not required for full water channel activity.

##### Loop D Mutants Do Not Relocalize to the Plasma Membrane in Response to Hypotonicity

**FIGURE 7.**
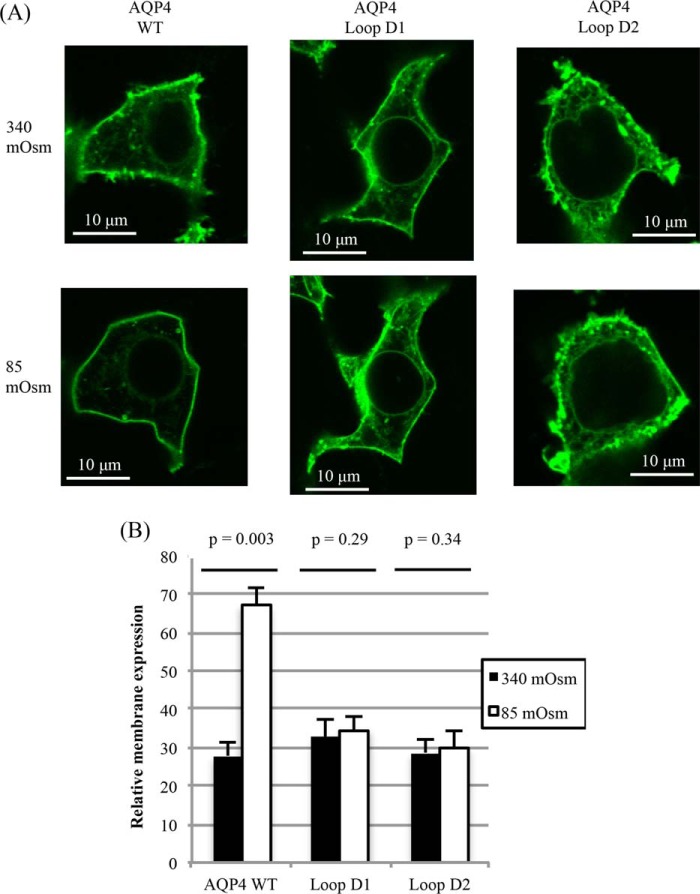
**Tonicity-induced translocation of non-tetrameric mutants.** *A,* representative fluorescence micrographs of HEK293 cells transfected with AQP4-GFP fusion proteins, before and after 30 s of exposure to hypotonic (85 mosmol) medium. *B,* relative membrane expression of AQP4-GFP fusion proteins before and after exposure to hypotonic medium. At least 4 cells per image were analyzed for each experimental repeat, *n* = 3. Data are presented as mean ± S.E.

We recently reported that AQP4 rapidly relocalizes to the plasma membrane from intracellular membranes in response to reduced extracellular tonicity (11) and that this phenomenon is true for other mammalian AQPs (7, 22). Despite wild-type water permeability and constitutive surface expression of the loop D mutants, D1 and D2, this response to hypotonicity was not observed for either. Relative membrane expression of wild-type AQP4-GFP imaged in live HEK293 cells increased from 27.9 ± 3.5 to 67.1 ± 4.5 (*p* = 0.003, *n* = 3) upon reduction of the extracellular tonicity to 85 mosmol, whereas the distribution of both the D1 and D2 mutants did not change significantly (*p* = 0.29 and *p* = 0.34 respectively, *n* = 3; Fig. 7).

##### Molecular Dynamics Simulations Suggest a Dynamic Network of Loop D Interactions

**FIGURE 8.**
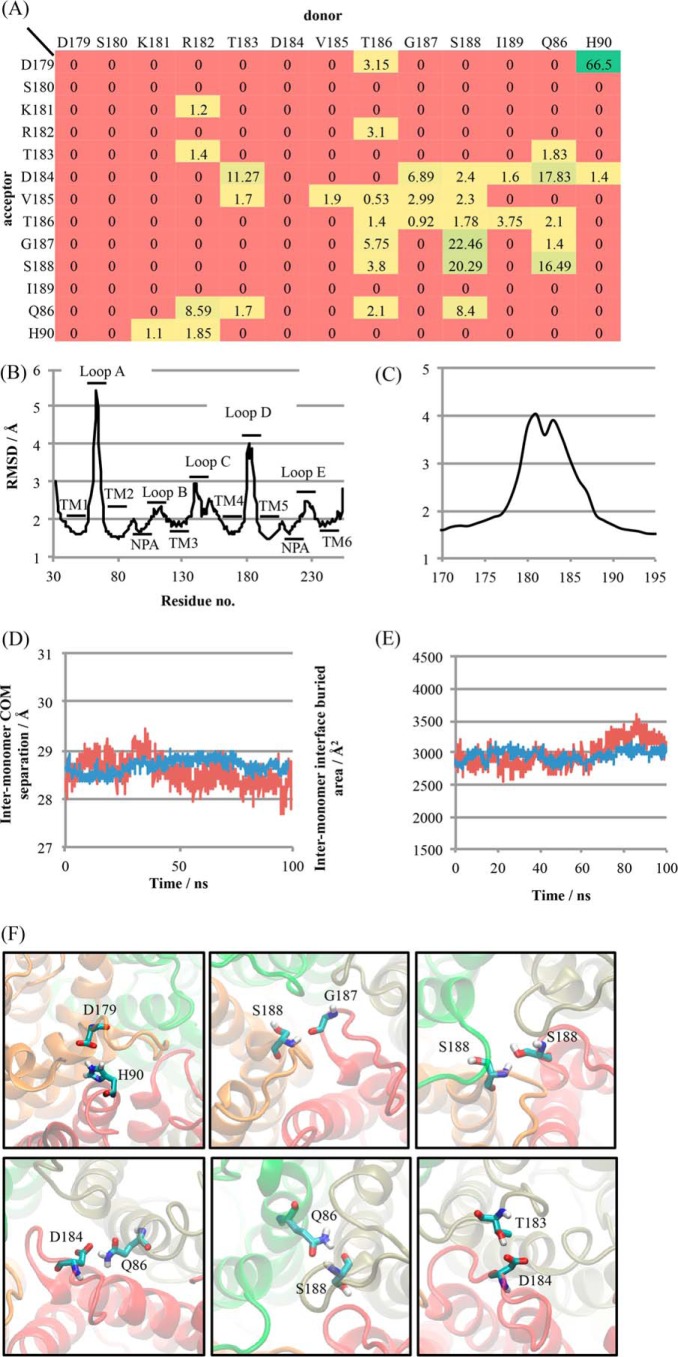
**A dynamic network of loop D hydrogen bonds in simulations of AQP4.** *A,* heat map showing percentage occupancy of loop D hydrogen bonds averaged over 4 monomers comprising a tetramer and over 5 independent 100-ns simulations. *B,* backbone heavy atom root mean square deviation (*RMSD*) of AQP4 residues, demonstrating the structural flexibility of loops A and D. RMSDs were calculated independently for each trajectory and averaged. *C*, close-up view of loop D RMSD. *D,* representative inter-monomer center of mass distance for a single interface (*red*) and averaged over the four interfaces of a tetramer (*blue*). *E*, representative inter-monomer buried area for a single interface (*red*) and averaged over the four interfaces of a tetramer (*blue*). *F*, snapshots of molecular dynamics trajectories in which the six most highly occupied inter-monomer hydrogen bonds involving loop D residues are occupied.

To investigate the contribution of loop D to AQP4 oligomerization we did five independent 100-ns molecular dynamics simulations of a hydrated AQP4 tetramer embedded in a 1-palmitoyl-2-oleoyl-*sn*-glycero-3-phosphocholine bilayer. We found a dynamic network of hydrogen bonds between loop D residues on adjacent monomers and also between loop D residues and both a glutamine (Gln^86^) and histidine (His^90^) residue at the bottom of TM2. On average, each monomer was involved in 3.8 ± 1.4 (mean ± S.D.) loop D hydrogen bonds with its two neighboring molecules at any given time, which is equivalent to 7.6 ± 2.8 loop D hydrogen bonds per tetramer. Every loop D residue apart from Ser^180^ was able to act as a hydrogen bond donor or acceptor with another loop D residue on one of the two adjacent monomers; 7 residues (Arg^182^, Thr^183^, Asp^184^, Val^185^, Thr^186^, Gly^187^, and Ser^188^) were found to have at least 3 different possible hydrogen bonding interactions. This network of interactions is represented as a heat map in Fig. 8*A*. Interactions involving Val^185^ and Gly^187^ were backbone hydrogen bonds. Both of these residues were able to act as hydrogen bond donors from the backbone amine group (Val^185^ to an adjacent Val^185^, and Gly^187^ to Asp^184^, Val^185^ and Thr^186^) and acceptors at the backbone carbonyl group (Val^185^ from an adjacent Val^185^ as well as Thr^183^, Thr^186^, Gly^187^, and Ser^188^, and Gly^187^ from Thr^186^, Ser^188^, and Gln^86)^. Loop D (as well as loop A) showed relatively large structural fluctuations (Fig. 8, *B* and *C*) despite maintaining an average of 3.8 ± 1.4 inter-monomer hydrogen bonding interactions. Representative snapshots showing the most highly occupied hydrogen bonds, His^90^-Asp^179^, Ser^188^-Gly^187^, Ser^188^-Ser^188^, Gln^86^-Asp^184^, Gln^86^-Ser^188^, and Thr^183^-Asp^184^ are shown in Fig. 8*F*. Loop D could potentially stabilize the AQP4 tetramer in one of two ways: by preventing monomers from drifting apart during rapid association/dissociation of the inter-monomeric TM interfaces, or by providing inter-monomer interactions that stabilize the tetramer in a more permanent way, preventing dissociation in the first place. In simulations we found that the average monomer-monomer center of mass separation was stable with a root mean square fluctuation of only 0.53 Å (*c.f*. typical carbon-carbon bond length of ∼1.5 Å) about a mean of 28.67 Å, and the inter-monomer buried (solvent-inaccessible) area was also stable, with a root mean square fluctuation of 230 Å^2^ about a mean of 2960 Å^2^. Representative traces are shown in Fig. 8, *D* and *E*. This suggests that there is no spontaneous dissociation/reassociation of the inter-monomer interfaces, at least on the time scale of these simulations (100 ns).

##### Loop D May Represent a Common Motif for Oligomeric Assembly Across the AQP Family

**FIGURE 9.**
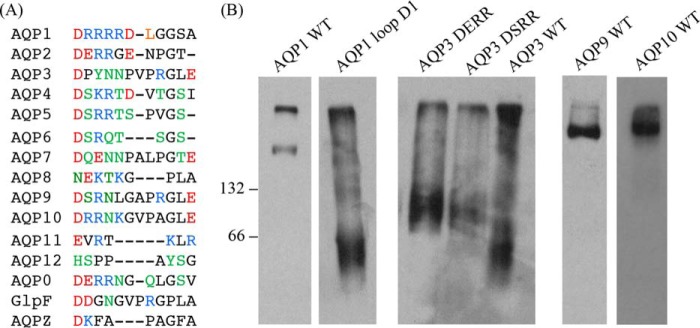
**Comparison of loop D between different human AQPs.** *A,* all 13 human AQPs as well as AQPZ and GlpF (both from *E. coli*) were aligned using Clustal Omega (EMBL-EBI). Acidic residues are colored *red*; basic, *blue*; and neutral residues able to form sidechain hydrogen bonds, *green. B,* BN-PAGE and Western blotting of wild-type and mutant AQPs 1 and 3 and wild-type AQPs 9 and 10. *66* and *132* represent BSA molecular weight markers.

To investigate whether our AQP4 data could be more widely applicable to other members of the AQP family, we performed a sequence alignment of the loop in all human AQPs, as well as the *E. coli* AQPs as comparison (Fig. 9*A*) using the Clustal Omega software (23). AQP1 contains a similar acidic-*X*-basic-basic motif to AQP4 in the first half of the loop (DRRRR *versus* DSKRT), so we made the D1 mutant in AQP1 (D158A/K159A/K160A/K161A/K162A). This motif is lacking in AQP3, therefore we introduced it via 2 different mutations, P181S/Y182K/N183R and P181E/Y182K/N183R (to give DSKRN and DEKRN *versus* the wild-type DPYNN). The D1 compound mutation in AQP1 caused a similar loss of oligomerization to that observed for AQP4. Both AQP3 mutations caused the protein to migrate primarily as a band with a molecular weight consistent with a dimeric species, whereas wild-type AQP3 appeared to migrate primarily as a monomer (Fig. 9*B*), consistent with previous studies on the oligomerization state of this AQP. Both AQP1 and AQP3 migrated as diffuse bands, consistent with glycosylation. PNGase F treatment was used to attempt deglycosylation. This appeared to be incompatible with the BN-PAGE experiments, because 1-h treatments with PNGase F at 37 °C caused AQP1 and −3 wild-type and mutants to form aggregates that did not migrate beyond the interface between the stacking and separating gels (data not shown). This may be due to increased temperature sensitivity of Triton X-100-solubilized AQPs. Finally, to investigate whether loss of AQP oligomerization is a general feature of the glyceroporin subfamily, we transfected HEK293 cells with AQPs 9 and 10 and subjected Triton X-100-extracted protein to BN-PAGE. Unlike AQP3, we found that AQPs 9 and 10 migrated exclusively as tetramers (Fig. 9*B*).

## Discussion

Our data show that the plasma membrane abundance of AQP4 in HEK293 cells is unaffected by the oligomeric state of the protein. This strongly suggests that tetrameric assembly is not required for AQP4 to be correctly trafficked through the endoplasmic reticulum and Golgi to the plasma membrane. It is possible that our mutants are trafficked as tetramers and then dissociate once inserted into the plasma membrane; the FRET data suggest that this is unlikely to be the case, although we cannot differentiate between particular intracellular compartments on the basis of this data, leaving open the possibility of differences in oligomerization state between different intracellular compartments. Our diffusion coefficients calculated from the FRAP data compare reasonably with other reports of diffusion of AQP-GFP constructs in mammalian cells (*e.g.* 5.7 × 10^−3^ μm^2^ s^−1^ for AQP2 (24), 3.1 × 10^−3^ μm^2^ s^−1^ for AQP1 (25)). We found a small, but statistically significant increase in diffusion coefficient for the mutants that were non-tetrameric in BN-PAGE. Although we have not estimated hydrodynamic radii for our constructs, the relationship D ∝ ln(1/R) derived by Saffman and Delbruck (26) suggests a hydrodynamic radius of 1.2 ± 1.0 nm for our non-tetrameric constructs (assuming *r* = 4 nm for a freely diffusing AQP4 tetramer), which is at least consistent with a loss of tetrameric assembly. We found no difference in the mobile fraction between the wild-type and D1 or D2 mutants (90 ± 6, 89 ± 4, and 92 ± 7%, respectively, n.s. *p* = 0.28), suggesting that there is no difference in the proportion of the protein involved in membrane anchoring interactions. Although we cannot rule out loss of an AQP4 binding partner as an alternative explanation of these data with absolute certainty, in combination with the cell surface BN-PAGE and FRET data it is highly suggestive of non-tetrameric mutant AQP4 molecules in the plasma membrane. Human AQP3 was reported to exist in all four possible oligomeric states (monomer, dimer, trimer, and tetramer) in the plasma membrane of erythrocytes (27). It may be that some or all members of the AQP family can be trafficked to the plasma membrane independently of their oligomerization state.

Our molecular dynamics simulations suggest a dynamic network of transient hydrogen bonds between the residues of loop D of adjacent monomers and also with two residues at the bottom of TM2. The fact that almost every residue involved in this network has several hydrogen bonding options may explain why none of the single alanine substitution mutations had an effect on tetrameric assembly. For example, the most highly occupied hydrogen bond that we found was His^90^-Asp^179^, which had 66.5% occupancy averaged over the four monomers and five trajectories. When this bond was not occupied, the Asp^184^ side chain was able to act as a hydrogen bond acceptor from the histidine imidazole NH group, and the Thr^186^ side chain hydroxyl group was able to act as a hydrogen bond donor to Asp^179^. These alternative interactions may be able to partially compensate for the loss of the Asp^179^-His^90^ interaction in the D179A and H90A single mutations. Asp^179^ had only one alternative interaction, whereas His^90^ had three. This may explain why the D179A mutation had a slight effect on oligomerization, whereas the H90A mutant did not. Interestingly, MD simulations have suggested that a histidine residue just beyond the bottom of TM2 in the intracellular loop B (H95) could modulate the channel open probability via spontaneous formation and dissociation of a hydrogen bond with a cysteine residue at the interface between TM4 and loop D (Cys^178^) (28). In our simulations, we did not observe this hydrogen bond. A recent study combining *in silico* and *in vitro* evidence also did not observe this hydrogen bond in simulations and suggested that His^95^ may indeed act as a channel gating residue, but by formation of a histidine protonation state-dependent salt bridge with a glutamate residue (Glu^41^), independent of Cys^178^ (29).

*S*-Nitrosylation of AQP11 at a cysteine residue in the extracellular loop E (Cys^227^) has been suggested to be required for AQP11 oligomeric assembly, with the C227S mutant showing reduced oligomeric assembly in mouse kidney (30). This supports the idea of a role for post-translational modification in AQP oligomerization. Loop D of AQP4 contains two sites predicted to be targets for post-translational modification. These are protein kinase sites at Ser^180^ and Ser^188^. Phosphorylation at Ser^180^ was suggested to reduce AQP4 water permeability via a gating effect in LLC-PK1 cells (31), but this was not supported by molecular dynamics simulations of AQP4-Ser(P)^180^ (32), and structural studies showed no difference between wild-type AQP4 and a phosphomimetic S180D mutant (33, 34). We used phosphomimetic (S180D, S188D) and phospho-blocking (S180A, S188A) mutations to investigate a potential role for post-translational modification in the AQP4 oligomeric assembly. We found that all four phosphorylation mutants were able to assemble into tetramers, suggesting that phosphorylation of loop D is not involved in tetrameric assembly of AQP4.

We recently reported that AQP4 in primary rat astrocytes and HEK293 cells rapidly relocalizes to the plasma membrane upon reduction of extracellular tonicity (11); this may involve changes in interactions between AQP4-containing vesicles and cytoskeletal elements as described by others (35, 36). Interestingly, neither of the mutants with reduced ability to tetramerize were able to relocalize in response to a hypotonic extracellular stimulus. It is possible that a binding partner of AQP4 involved in the translocation response recognizes an epitope formed by the interface of several AQP molecules within a tetramer, and that disrupting tetrameric assembly disrupts this epitope. Although the C-terminal PKA phosphorylation site (Ser^276^), which controls this response is ∼100 residues away in the primary sequence, it may be that phosphorylation of this residue causes a conformational change in the large (∼70 residue) C-terminal tail of AQP4, which allows an AQP4-binding protein to bind to the intracellular face of AQP4 including loop D. Structural data for AQPs are routinely collected after cleaving the C terminus at the bottom of TM6 to aid crystallization and high-resolution structure determination (37). This is true for AQP4 and in the structure that we used to identify mutagenic targets and as input for our simulations, the protein was truncated at the bottom of TM6, at residue 254 of 323 (17). This makes it difficult for us to make any concrete predictions about interactions of the AQP4 C terminus, especially given its large size.

Based on a sequence alignment of loop D, we made mutants of human AQPs 1 and 3. Wild-type AQP1 existed as a tetramer when extracted from HEK293 membranes using Triton X-100, whereas AQP3 existed primarily as a monomer, both of which are in agreement with previous reports (27, 38). Mutating the first five residues of AQP1 loop D to alanine (which rendered AQP4 primarily monomeric) caused the protein to migrate primarily as a monomer in BN-PAGE. Introducing the conserved acid-*X*-base-base motif (present in loop D of both AQPs 1 and 4) into AQP3 caused it to migrate primarily as a dimer. It has been suggested that lack of oligomerization may have a role in controlling substrate selectivity of the glyceroporin subfamily (39), which consists of AQPs 3, 7, 9, and 10 in humans. However, we find that, unlike AQP3, both AQPs 9 and 10 both exist exclusively as tetramers when extracted from HEK293 membranes. This suggests that solute permeability of AQPs is not correlated with the oligomeric state.

Previous mutational analysis of AQP1 found an extracellular motif consisting of an aspartate residue (Asp^185^) at the top of TM5 that can interact with a lysine residue (Lys^51^) at the top of TM2 to increase tetramer stability in detergent (40). Whether this had an effect on native protein in live cells was not clear. The S205D mutant of an insect AQP (AQPcic) was shown to exist in a primarily monomeric state when extracted from yeast and *Xenopus* oocyte membranes, whereas the wild-type was tetrameric (41, 42). This mutant had a complete loss of water channel function, and it is not clear whether this was due to subtle changes in structure leading to a gating effect, gross misfolding, loss of surface expression, or a direct result of the loss of oligomerization through loss of monomer-monomer interactions that stabilize the open state of the pore. This makes interpretation of this result very difficult.

In summary, we show that loop D of AQP4 forms a hub for a dynamic network of interactions that stabilize the AQP4 tetramer, both when solubilized using non-ionic detergent and in living mammalian cells. Tetrameric assembly is not required for either endoplasmic reticulum-to-Golgi-to-plasma membrane trafficking or water channel activity. Mutants with reduced tetrameric assembly are unable to relocalize in response to tonicity changes, which may reflect a requirement for tetramerization in the regulation of AQP relocalization or key protein-protein interactions mediated by loop D of AQP4. We conclude that loop D interactions may represent a conserved mechanism for controlling oligomerization across the AQP family.

## Author Contributions

A. C. C., P. K., M. T. C., and R. M. B. designed all experiments. P. K. performed and analyzed all experiments and prepared figures. P. K. and A. C. C. drafted the manuscript. R. M. B. and M. T. C. critically revised the manuscript. All authors approved the final version of the manuscript.

## Footnotes

3 The abbreviations used are:

AQP: aquaporin
FRET: Forster resonant energy transfer
FRAP: fluorescence recovery after photobleaching
TM: transmembrane domain
MDCK: Madin-Darby canine kidney cells
BN-PAGE: blue native-PAGE
bis-tris: 2-[bis(2-hydroxyethyl)amino]-2-(hydroxymethyl)propane-1,3-diol
Tricine: *N*-[2-hydroxy-1,1-bis(hydroxymethyl)ethyl]glycine
ANOVA: analysis of variance.
